# Fracture Mapping in High-Energy Chest Trauma

**DOI:** 10.3390/jcm13206127

**Published:** 2024-10-14

**Authors:** Shuhuan Li, Duo Sun, Chu Wang, Pan Hu, Feifei Jin, Wei Huang

**Affiliations:** 1 Trauma Treatment Center, Peking University People’s Hospital, Beijing 100044, China; lshuhuan@126.com (S.L.); feifeijin92@163.com (F.J.); 2 Key Laboratory of Trauma Treatment and Neural Regeneration, National Center for Trauma Medicine, Ministry of Education, Peking University, Beijing 100044, China

**Keywords:** high-energy chest trauma, rib fractures, 3D fracture mapping, fracture-frequency heat maps

## Abstract

**Background**: High-energy chest trauma often results in rib fractures and associated chest injuries. This study explored fracture distribution patterns in high-energy chest trauma, using three-dimensional (3D) fracture mapping technology. **Methods**: This retrospective study analyzed cases of high-energy chest trauma with rib fractures treated at a Level 1 Trauma Center, from February 2012 to January 2023. Specifically, 3D computed tomography (CT) was used to reconstruct rib fractures and create fracture-frequency heat maps, analyzing the influence of other thoracic fractures on rib fracture distribution. **Results**: Rib fractures were frequently found in the anterior and posterior thoracic areas. On average, patients sustained 7 ± 3.87 rib fractures, with clavicle fractures in 25.5% and scapular fractures in 19.6% of cases. Scapular fractures led to more posterior rib fractures, while sternal fractures were associated with more anterior rib fractures. Clavicle fractures were linked to fractures of the first to third ribs. **Conclusions**: Rib fractures in high-energy chest trauma occurred most often in the anterior and posterior regions. Fractures of the scapula and sternum influence the positioning of the fracture lines. Clavicular fractures are associated with a higher incidence of upper rib fractures. These findings can help inform surgical decisions and complication management.

## 1. Introduction

Chest trauma accounts for 10–15% of all trauma cases and, over the past decade, the incidence rate of rib fractures per 100,000 people has increased by 52%. Rib fractures comprise approximately 10% of all trauma-related injuries, with an overall mortality rate of 10–12% [1,2,3,4,5]. Blunt chest trauma frequently results in rib fractures, commonly associated with pneumothorax, hemothorax, and pulmonary contusion. High-energy trauma often causes rib fractures to coincide with fractures of the clavicle, scapula, and other thoracic bones [6]. Clavicle fractures constitute 2.6–4% of all fractures, with approximately 81% occurring in the middle third of the clavicle [7,8]. According to the National Trauma Data Bank in the United States, the incidence of scapular fractures has doubled over the past decade (from 1% to 2.2%), with high-energy motor vehicle accidents remaining a primary cause [9]. Sternal fractures, though rare, result from high-energy mechanisms affecting the chest wall [10,11]. Multiple injuries may include, but are not limited to, these types of fractures. Recent evidence suggests that compared to conservative treatment, surgical fixation of rib fractures, especially flail chest and multiple rib fractures, can significantly reduce mortality rates [12]. Rib fixation surgery can restore pre-injury activity levels in patients with flail chest or multiple rib fractures, providing significant long-term benefits [13]. Detailed descriptions of rib fracture locations and patterns of other thoracic fractures as a result of high-energy chest trauma are crucial for understanding the underlying mechanisms and for preoperative planning, thereby facilitating more effective research and treatment strategies.

The Orthopaedic Trauma Association/AO (OTA/AO) introduced the rib fracture classification system in 2018, establishing an initial typology for rib fractures [14]. In 2019, the Chest Wall Injury Society (CWIS) developed a rib fracture classification system, categorizing fractures into three anatomical sectors: anterior, lateral, and posterior. However, no final consensus was reached on the precise definition of the boundaries between these sectors [15]. In 2023, the CWIS, in collaboration with the American Society of Emergency Radiology (ASER), updated the taxonomy, emphasizing the ongoing challenges in regard to boundary definitions and highlighting the need for further research [16].

Fracture mapping technology effectively overlays fracture models on a complete model template and then extracts fracture lines in order to display typical fracture characteristics [17]. The three-dimensional fracture heat map generated from 3D fracture line mapping provides a more comprehensive understanding of fracture patterns and morphology. This technology has been successfully applied to analyze fracture features at various anatomical sites, such as the scapula, tibial plateau, calcaneus, and acetabular fractures [17,18,19,20,21]. This technique allows the distribution and morphology of rib fractures caused by high-energy trauma to be understood, providing critical information for further typological research and treatment. To date, few studies have been conducted on the precise fracture patterns and injuries associated with rib fractures caused by high-energy chest trauma. This study explored the application of fracture-frequency maps to traumatic chest injuries, visualizing the location and patterns of rib fractures and the impact of other thoracic injuries on rib fractures, offering insight into the characteristics of high-energy chest trauma and its clinical significance. Also, fracture maps may be used for rib fracture classification.

## 2. Materials and Methods

### 2.1. Study Population

This retrospective analysis reviewed high-energy chest trauma cases treated at a Level 1 Trauma Center, Trauma Treatment Center, Peking University People’s Hospital, Beijing, China, between February 2012 and January 2023. This study included 102 cases of chest trauma, with rib fractures that met the high-energy injury criteria. High-energy mechanisms were defined as significant kinetic forces and were categorized as follows: motor vehicle accidents (motor vehicle > 65 km/h), which included vehicle-to-vehicle collisions, pedestrian-to-vehicle collisions, electric bicycle-to-vehicle collisions, motorcycle-to-vehicle collisions, and bicycle-to-vehicle collisions; and non-vehicular high-energy injuries, which included motorcycle accidents (>45 km/h); falls from a height greater than 3 m; heavy object impact; mechanical, crush, and biogas explosion injuries [22]. 

The data collected included the location and type of rib fractures (unilateral or bilateral), the associated thoracic injuries (such as clavicular, scapular, thoracic vertebral, and sternal fractures), and other relevant clinical characteristics. The information extracted from the patients’ electronic medical records included sex, age, type of injury, associated injuries, and treatments administered.

### 2.2. Rib Fracture Mapping

The rib fractures were three-dimensionally (3D) reconstructed and the fracture lines were plotted to create fracture-frequency heat maps. The patients’ computed tomography (CT) data, in Digital Imaging and Communications in Medicine (DICOM) format, were imported into the medical image processing software (Mimics) version 21.0 (Materialize NV, Leuven, Belgium) to reconstruct the 3D model [19]. A grayscale range of 155–1594 Hounsfield units (HUs) was set as the threshold for rib segmentation. For bones that were incompletely segmented within this HU range (e.g., in cases of osteoporosis) or where non-bony tissue was not fully excluded, the HU range was adjusted to determine the optimal threshold. The cortical bone window was selected to segment the entire rib area, separate the bony anatomical structures, and individually segment and reconstruct the fracture endpoints. The overall segmentation and reconstruction range started at the intersection of the rib with the spine (costovertebral joint) and extended to the fracture endpoint, then from the fracture endpoint to the boundary between the rib shaft and the anterior end segment (costochondral junction), which is the endpoint of the bony rib. The anterior end segment is defined as the costochondral cartilage, representing the anterior part of the rib. The rib shaft is defined as the bony portion that connects the anterior and posterior end segments, forming the main middle section of the rib. The posterior end segment is defined as the portion between the rib tubercle and the rib shaft, specifically extending from the costovertebral joint to the tip of the transverse process (costotransverse articulations). These three segments together form a continuous structure, spanning from the spinal connection at the posterior end to the anterior cartilaginous junction. The 3D reconstruction and measurement scope of the rib includes the posterior end segment and the rib shaft. The rib ends and segments were described according to the 2018 AO (OTA/AO) classification [14]. 

**Figure 1:**
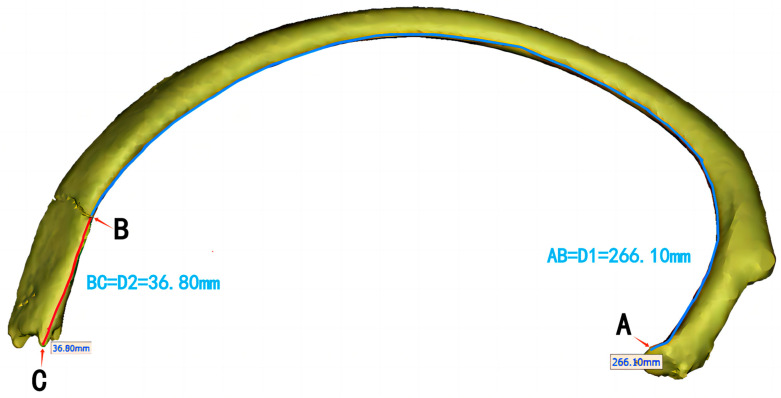
Schematic diagram of rib fracture line measurement. The junction between the rib and spine (costovertebral joint) is marked as A, the fracture site as B, and the boundary between the rib shaft and the anterior end segment (costochondral junction) as C. The distance from A to B is denoted as D1 and the distance from B to C is denoted as D2. The percentage of rib fractures along the ribs was calculated as follows: D1/(D1 + D2) × 100. The schematic diagram illustrates that the percentage of rib fractures was 266.10/(266.10 + 36.80) × 100% = 87.85%.

Using the ‘distance over the surface’ function in Mimics, the position of the rib fracture line was measured along a curve, and the total length of the rib was measured to calculate the percentage of the rib length occupied by the fracture line (Figure 1). The measured model was saved as an STL file for subsequent fracture line plotting. For cases with poor fracture alignment or displacement, the 3D-reconstructed STL file was imported into E-3D software Version 19.3 (Central South University, Changsha, China), where the ‘block separation’ tool was used to select both sides of the fracture endpoints. The fracture reduction function was then employed to realign significantly displaced fractures, restoring the rib’s normal anatomical arc and continuity. The adjusted model was exported in STL format and re-imported into Mimics for further measurement of the fracture line’s percentage location.

A healthy adult rib was used as the standard template. Using the E-3D software, the fracture lines were plotted on the standard template, according to the measured percentage positions, using the curve measurement and curve plotting functions. The measured fracture percentage positions of the rib fracture lines were plotted on a standard template, using the curve plotting function, and 3D fracture-frequency heat maps were generated based on the plotted fracture lines. In the 3D heat maps, different colors represent different fracture frequencies. The location of the rib fractures, including the anterior, lateral, and posterior regions, was based on the CWIS 2020 consensus [15].

### 2.3. Statistical Analysis

The statistical methods used included descriptive statistics and analysis of the rib fracture data using sociodemographic data, including the percentage of rib fractures, the count of fractures, and the number of fracture occurrences. Descriptive analysis of the quantitative variables included the mean, standard deviation, median, minimum, and maximum values. Categorical variables, such as age, sex, injury mechanisms, associated injuries, and treatment modalities, were reported as frequencies and percentages. Patient characteristics and fracture measurements were summarized using the mean and standard deviation or proportions. Comparisons between the two groups were made using appropriate tests based on the data distribution; grouped t-tests were used for data following a normal distribution and the Wilcoxon rank sum test was employed for data that did not follow a normal distribution. Categorical indicators were analyzed using the Chi-square test or Fisher’s exact test, with all statistical tests being two-sided, unless specified otherwise, and a *p* < 0.05 was considered statistically significant. All statistical analyses were performed using SPSS software version 26.0 (IBM Corp., Armonk, NY, USA). 

## 3. Results

We excluded a total of 33 cases, including 14 cases assessed only in outpatient or emergency settings without hospitalization, 11 with incomplete CT data or artifacts, and 8 with a CT slice thickness greater than 2.5 mm. A total of 102 patients met the inclusion criteria: the average age was 54.5 ± 13.2 years; 74 were male (72.5%) and 28 were female (27.5%) (Table 1). The most prevalent mechanism of injury was motor vehicle accidents, accounting for 74 (72.5%) cases. This category included vehicle–vehicle collisions (50 cases, 49.0%), pedestrian versus vehicle collisions (6 cases, 5.9%), motor vehicle versus electric bicycle collisions (12 cases, 11.8%), motor vehicle versus motorcycle collisions (2 cases, 2.0%), and bicycle versus motor vehicle collisions (4 cases, 3.9%). Among the other injury mechanisms, falls from a height greater than 3 m were the most common, accounting for 15 cases (14.7%) (Table 1).

The most frequent associated injuries were pulmonary contusions (47 cases, 46%), extremity fractures (38 cases, 37.3%), pneumothorax (38 cases, 37.3%), abdominal injury (32 cases, 31.4%), pelvic fracture (29 cases, 28.4%), and hemothorax (28 cases, 27.5%); 7 patients (6.9%) had flail chest (Table 2). Other thoracic fractures, excluding rib fractures: clavicle fractures occurred in 26 patients (25.5%), with 19 on the right side and 7 on the left; scapular fractures occurred in 20 patients (19.6%), with 9 on the right side and 11 on the left side; simultaneous clavicle and scapular fractures occurred in 7 patients (6.9%); thoracic vertebral fractures occurred in 11 patients (10.8%); and sternal fractures occurred in 4 patients (3.9%). The complete data can be found in Table S1. Among the patients with high-energy chest trauma, 36.3% underwent thoracic surgery. Apart from the thorax, the most common surgical site was the extremities, accounting for 26.5% of cases (Table 2).

**Figure 2:**
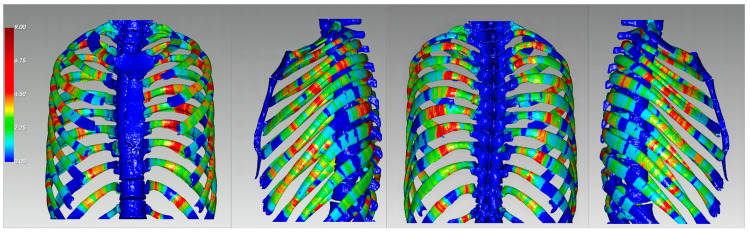
3D Reconstruction of rib fracture heat maps. Rib fractures were reconstructed three-dimensionally with the fracture lines overlaid on a template to create 3D heat maps. These maps show a heat map of the frequency of rib fracture lines after thoracic trauma. A heat map is shown of anterior, left, posterior, and right views (from left to right). Blue indicates areas with fewer fracture lines, red indicates areas with more fracture lines, and the gradients from blue to green and yellow to red indicate an increasing frequency of fracture lines.

**Figure 3:**
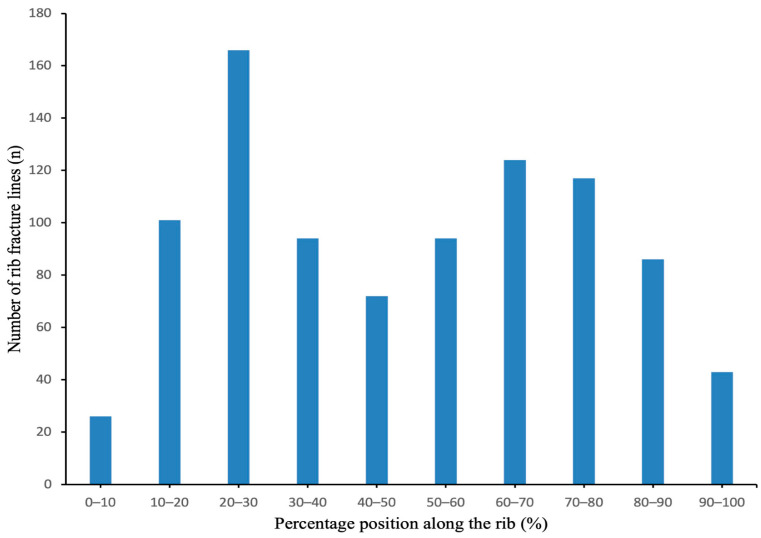
Distribution of rib fracture lines by percentage position along the rib.

We generated rib fracture-frequency and heat maps (Figure 2), indicating that the most common rib fracture locations were the anterior and posterior regions of the thoracic cavity, with fracture lines mainly concentrated in ribs 3–7, where each rib frequently exhibited multiple fractures. Rib fracture lines were most commonly located in the 20–30% and 60–70% segments of the rib, with 166 and 124 fractures, respectively. The 10–20% and 70–80% segments also had relatively high fracture counts, with 101 and 117 fractures, respectively. In contrast, the fracture frequency was lower in both the 0–10% and 90–100% regions of the rib (Figure 3). These findings indicate a higher concentration of fractures in the anterior and posterior sections of the rib, suggesting that these areas may be more susceptible to fractures in high-energy trauma. 

A total of 4 patients sustained a single unilateral rib fracture, while 47 patients sustained bilateral rib fractures, and 90 patients sustained two or more consecutive rib fractures. The average number of rib fractures was 7 ± 3.87; the average number of fracture events was 9.05 ± 5.83. Rib fractures on the left and right sides accounted for 52.00% and 48.00% of cases, respectively. The fractures of ribs 3–7 each exceeded 10%, with percentages of 12.03%, 13.11%, 14.08%, 11.70%, and 12.13%, respectively, totaling 63.05%. In contrast, the 11th and 12th ribs exhibited the lowest fracture rates at 2.82% and 0.65%, respectively (Table 3). 

The average rib fracture location was 49.60%, with a standard deviation of 25.35%. In patients with concomitant clavicle fractures, the mean location of the rib fractures was 50.53%, with a standard deviation of 26.51%, indicating that clavicle fractures did not significantly affect the position of the rib fractures (*p* = 0.513). In patients with concomitant scapular fractures, the fracture line was positioned more posteriorly and closer to the spine, with a mean location of 43.58% and a standard deviation of 26.19% (*p* < 0.001). For concomitant thoracic vertebral fractures, the mean location was 48.49%, with a standard deviation of 24.93%, showing that these fractures did not significantly affect the location of the rib fractures (*p* = 0.728). In concomitant sternal fractures, the fracture line was located more anteriorly (61.32%), with a standard deviation of 26.66% (*p* = 0.003) (Table 4). Further analysis revealed a significant difference in the prevalence of first-to-third rib fractures, with a rate of 92.31% when clavicle fractures were concomitant, compared to 69.74% without clavicle fractures (*p* = 0.001) (Table 5). 

## 4. Discussion

In this study, we used rib fracture mapping technology to precisely describe the distribution and patterns of rib fractures in patients with high-energy chest trauma. Our fracture maps revealed that fractures predominantly occurred between the third and seventh ribs, with widespread fracture lines in the anterior and posterior regions of the ribs. Fracture lines were most commonly located in the 20–30% and 60–70% segments of the rib. High-energy chest injuries often involve concurrent fractures of the clavicle, scapula, thoracic vertebrae, or sternum. We also explored the influence of different types of associated fractures on the location and pattern of rib fractures. Concurrent clavicle fractures are commonly associated with fractures of the upper ribs, reflecting the directional force towards the upper chest, resulting in more frequent fractures of the upper ribs. Rib fractures associated with scapular fractures tend to be closer to the spine, indicating that ribs below the scapula are more susceptible to scapular fractures. Concurrent sternal fractures typically lead to rib fractures closer to the sternum, likely related to the mechanics of the anterior impact. These findings have direct practical value for surgical planning and treatment strategies.

Our findings indicate that the average number of rib fractures in high-energy injuries is 7 ± 3.87, with an average incidence of 9.05 ± 5.83 fractures per case, which significantly differs from the findings by Pines G et al. [23], who reported an average of 3.82 ± 1.68 fractures per person, possibly due to their inclusion of more low-energy injuries, leading to a lower average number of rib fractures. Our results are close to those that Christian et al. [24] reported, with an average of 9.8 fractures per case. Flail chest was observed in 6.9% of patients, consistent with other studies reporting a prevalence of 5.8–8% [1,6,25]. We found that fractures of the third-to-seventh ribs each accounted for more than 10% of cases, totaling 63.05%, corroborating other reports that reported that over 50% of rib fractures occur in the fourth-to-seventh ribs [24,25]. These studies confirm that high-energy chest trauma frequently leads to extensive rib fractures.

Our study is the first to precisely measure rib fracture lines using 3D reconstruction, generate detailed fracture heat maps, and further analyze the specific impact of concomitant fractures on the location of fracture lines, which is rare in previous research. Our findings conclude that rib fractures commonly occur in the anterior and posterior regions of the thorax, with fracture lines most commonly located in the 20–30% and 60–70% segments of the rib. This pattern is likely due to the concentration of impact forces on the outer sections of the ribs, making them more prone to fracture [24]. Thomas CN et al. [25] conducted a study using a different 2D CT rib unfolding technique. They manually drew rib fracture lines on 2D unfolded ribs and generated rib fracture heat maps, focusing on a younger population. They obtained similar results; however, the injuries in their population were less severe, accounting for only half of the rib fractures observed in our study. Additionally, they did not analyze the factors related to the distribution of rib fractures.

Rib fractures are the most common injury in chest trauma, followed by pneumothorax, extremity fractures, cranial injuries, and clavicle fractures [26]. We further explored the association between other thoracic fractures and the distribution of rib fracture lines, finding that other chest fractures significantly influence the location of rib fracture lines. In our study, scapular fractures notably affected the endpoints of rib fractures, positioning them closer to the spine, likely because of the external forces acting on the area of the scapular fracture, causing the underlying ribs to fracture. Other studies indicate that 58.1% of scapular fractures occur with ipsilateral rib fractures, predominantly in the third-to-sixth ribs below the scapula [27]. Research by LaRoque MC et al. [28] involved the development of a sternum fracture map, revealing that over 80% of sternum fractures are associated with rib fractures; however, they did not analyze the influence of the sternum on rib fracture line locations. Our further study of sternum fractures found that such injuries likely lead to rib fractures closer to the midline.

Regarding the relationship between clavicle fractures and rib fractures, our findings showed that 25.5% of cases with high-energy chest trauma involving rib fractures also included clavicle fractures, which was significantly higher than the 14–18.8% reported by Sweet et al. [29]. This increase may be due to the higher incidence of clavicle fractures in high-energy chest injuries. Our study also indicated that clavicle fractures tend to occur with upper rib fractures (first-to-third ribs), providing important insights for clinicians in order to assess the complexity of thoracic injuries. Additionally, using more accurate 3D reconstructed CT data allowed for precise quantification of the impact of other thoracic fractures on rib fracture lines, aiding the formation of a better understanding of the biomechanical properties and mechanisms of rib fractures.

Motor vehicle accidents most commonly cause rib fractures [1,3]. Our results showed that the most frequent mechanism of injury was motor vehicle accidents (72.5%) and pulmonary contusion (46%) was the most common thoracic complication. Research by Peek et al. [30] found that the most common thoracic complication was pneumothorax (37.2%), which may be attributed to our study population, which excluded patients with low-energy injuries, who predominantly suffered pulmonary contusions due to blunt chest trauma. In comparison to the study by Lin et al. [26], their findings indicated that the most commonly associated injury in patients with traumatic rib fractures was extremity fracture (26.7%). The incidence of extremity fractures in our study varied slightly, and our rates of extremity fractures and cranial injuries were significantly higher than theirs, likely because our center treats high-energy trauma patients primarily involved in severe motor vehicle crashes, leading to a higher frequency of multiple fractures. The most common surgical procedures found in our study were thoracic surgery (36.3%) and extremity surgery (26.5%). In contrast, other studies have reported that the most frequent surgical procedures were thoracic closed drainage (22.7%) and extremity surgery (20.6%). The likely reason for this difference is that our cases involved high-energy trauma patients, most of whom had multiple injuries, rib fractures, and other thoracic fractures; hence, our findings included a greater number of thoracic and extremity surgeries [26]. 

The importance of surgical intervention for rib fractures has been emphasized, with recent studies indicating that early surgical fixation can improve the prognosis of polytrauma patients with rib fractures [31]. Surgical fixation of rib fractures, particularly in flail chest cases, reduces the incidence of respiratory failure, diminishes mechanical ventilation requirements, and lowers the occurrence rate of pneumonia [32]. Polytrauma patients with multiple rib fractures and associated pulmonary contusion benefit from early rib fracture surgical stabilization within 72 h. This intervention has been shown to reduce the duration of hospitalization and the length of stay in the intensive care unit (ICU), as well as the rates of unplanned endotracheal intubation and unplanned ICU admission [33]. Surgical stabilization of multiple rib fractures reduces the duration of mechanical ventilation and lowers the incidence of pneumonia [34]. The application of rib fracture mapping technology enhances the accuracy of diagnosing rib fractures in high-energy chest trauma cases. This technology enables physicians to predict potential complications and better assess the necessity of surgery and the choice of surgical approach, potentially reducing the risk of complications, shortening hospital stays, and improving the patient’s quality of life.

The results of this study highlight the importance of applying fracture mapping technology to chest injuries to understand and predict clinical diagnosis and treatment. Applying fracture mapping technology and precise fracture line measurements in treating high-energy chest trauma provides new directions for future research and therapy. These findings can assist clinical physicians in accurately identifying the location of rib fractures and also hold significant clinical significance in regard to formulating effective treatment strategies, providing valuable references for the future classifications of rib fractures and associated injuries.

This study has some limitations. First, the limited sample size could be expanded to obtain more comprehensive data. Additionally, the creation of fracture maps relies on high-quality CT imaging data, and the quality and accuracy of data processing directly affect the accuracy of the results. The lack of separate classifications for different age and sex groups and potential anatomical differences among the individuals may affect the universality and applicability of the fracture maps. Furthermore, the study did not analyze the correlation between rib fracture distribution and clinical prognosis or propose new classifications. The absence of certain data, such as body weight and vital signs, also limits the study’s comprehensiveness. Future research should incorporate more individualized clinical data, as these parameters may be correlated with the location and distribution of rib fractures and could provide stronger support for personalized treatment in chest trauma. In addition to incorporating clinical data, future research should further validate and improve the rib fracture mapping technology, expand the sample size, conduct multicenter studies, and establish new classifications for rib fractures and associated injuries. Furthermore, exploring the possibility of integrating rib fracture mapping technology with other diagnostic tools, such as machine learning-based prognostic assessment tools for rib fractures, to predict patient recovery paths and potential complications based on specific rib fracture data (such as location, number, and type), can improve the accuracy and efficiency of diagnosis and treatment planning. These findings help to establish a better understanding of the characteristics of rib fracture injuries and provide a scientific basis for developing more effective treatment strategies. 

## 5. Conclusions

In high-energy chest trauma, rib fractures are numerous, with the most common locations being the anterior and posterior regions. Fractures of the scapula and sternum influence the positioning of fracture lines. Clavicular fractures are associated with a high incidence of upper rib fractures. Data visualization and the assessment of precise patterns of rib fractures offer possibilities in terms of predicting complications, assessing the necessity of surgery, and choosing the appropriate surgical approach.

## Figures and Tables

**Table 1:** Chest trauma demographic data.

Variable	N = 102
Age—mean ± SD, yr	54.5 ± 13.2
Male (n, %)	74 (72.5)
Female (n, %)	28 (27.5)
Mechanism of Injury	n(%)
Motor vehicle accidents	74 (72.5)
Vehicle versus vehicle	50 (49.0)
Pedestrian versus vehicle	6 (5.9)
Electric bicycle versus vehicle	12 (11.8)
Motorcycle versus vehicle	2 (2)
Bicycle versus vehicle	4 (3.9)
Motorcycle accident	7 (6.9)
High fall (>3 m)	15 (14.7)
Heavy object impact injury	3 (2.9)
Mechanical injury	1 (1.0)
Crush injury	1 (1.0)
Biogas explosion injury	1 (1.0)

SD: standard deviation.

**Table 2:** Thoracic injuries with associated injuries and treatments.

Injury Type	n (%)
Thoracic Fracture	
Rib fracture	102 (100)
Clavicle fracture	26 (25.5)
Scapular fracture	20 (19.6)
Thoracic vertebra fracture	11 (10.8)
Sternum fracture	4 (3.9)
Other Associated Injury	
Intracranial hemorrhage	23 (22.5)
Cranial fracture	10 (9.8)
Cerebral contusion and laceration	11 (10.8)
Pneumothorax	38 (37.3)
Hemothorax	28 (27.5)
Hemopneumothorax	23 (22.5)
Pulmonary contusion	47 (46)
Atelectasis	13 (12.7)
Flail chest	7 (6.9)
Abdominal injury	32 (31.4)
Cervical vertebra fracture	6 (5.9)
Lumbar vertebra fracture	18 (17.6)
Extremity fracture	38 (37.3)
Pelvic fracture	29 (28.4)
Hemorrhagic shock	18 (17.6)
Aortic dissection	2 (2.0)
Femoral artery injury	2 (2.0)
Pulmonary embolism	1 (1.0)
Cardiac contusion	2 (2.0)
Treatments	
Thoracic surgery	37 (36.3)
Cranial surgery	4 (3.9)
Abdominal surgery	7 (6.9)
Spine surgery	8 (7.8)
Extremity surgery	27 (26.5)
Pelvic surgery	15 (14.7)
Vascular surgery	3 (2.9)

**Table 3:** Number of rib fractures on each rib.

Number of Rib Fractures	Left (n)	Right (n)	Total	Percentage (%)
1st Rib	20	19	39	4.23
2nd Rib	37	41	78	8.45
3rd Rib	56	55	111	12.03
4th Rib	65	56	121	13.11
5th Rib	68	62	130	14.08
6th Rib	55	53	108	11.70
7th Rib	56	56	112	12.13
8th Rib	45	42	87	9.43
9th Rib	36	32	68	7.37
10th Rib	23	14	37	4.01
11th Rib	15	11	26	2.82
12th Rib	4	2	6	0.65
Total	480	443	923	100
Percentage (%)	52	48	100	

**Table 4:** Effects of various thoracic fractures on the position of rib fracture lines.

Fracture Type	Position of Rib Fracture Lines	Standard Deviation	U Statistic	*p*-Value
Clavicle Fracture				
No	49.21%	24.87%		
Yes	50.53%	26.51%	90,567.000	0.513
Scapular Fracture				
No	51.03%	24.95%		
Yes	43.58%	26.19%	55,165.000	<0.001 *
Thoracic Vertebrae Fracture				
No	49.73%	25.41%		
Yes	48.49%	24.93%	41,342.000	0.728
Sternum Fracture				
No	49.06%	25.17%		
Yes	61.32%	26.66%	22,530.000	0.003 *
All Rib Fractures	49.60%	25.35%		

* *p* < 0.05. Note: This table analyzes the impact of different thoracic fractures (e.g., clavicle, scapular, thoracic vertebrae, and sternum fractures) on the average percentage position of rib fracture lines. U statistics and *p*-values were used to assess whether each fracture type significantly affects rib fracture line positions. The results show that scapular and sternum fractures significantly affect rib fracture line positions, while clavicle and thoracic vertebrae fractures do not.

**Table 5:** Impact of clavicle fractures on 1st–3rd rib fracture rates.

Clavicle Fracture	Fracture Rate of the First-to-Third Ribs	Chi-Square Statistic (χ^2^)	*p*-Value
No	69.74%		
Yes	92.31%	10.489	0.001 *

* *p* < 0.05.

## Data Availability

The data presented in this study are included within the manuscript and its Supplementary Information Files (Table S1).

## Supplementary Materials

The following supporting information can be downloaded at: https://www.mdpi.com/article/10.3390/jcm13206127/s1.
